# Complete Genome Sequences of Beijing and Manila Family Strains of *Mycobacterium tuberculosis*

**DOI:** 10.1128/genomeA.01135-14

**Published:** 2014-11-13

**Authors:** Xuehua Wan, Lishi Qian, Shaobin Hou, Kevin P. Drees, Jeffrey T. Foster, James T. Douglas

**Affiliations:** a Advanced Studies in Genomics, Proteomics and Bioinformatics, University of Hawaii, Honolulu, Hawaii, USA; b Department of Microbiology, University of Hawaii, Honolulu, Hawaii, USA; c Department of Molecular, Cellular and Biomedical Sciences, University of New Hampshire, Durham, New Hampshire, USA

## Abstract

The majority of isolates from tuberculosis patients in Hawaii arrive through the immigration of infected individuals from the western Pacific. We report here on the annotated complete genomes of two strains of *Mycobacterium tuberculosis* from the two main lineages/families in Hawaii, Beijing and Manila.

## GENOME ANNOUNCEMENT

Hawaii leads the United States with one of the highest rates of tuberculosis, at 8.4/100,000 people in 2012 (1). Most cases come from immigrants from the western Pacific, including countries, such as the Philippines, China, and South Korea, as well as the Pacific Islands, constituting approximately 120 cases/year (http://health.hawaii.gov/tb/data-statistics/). *Mycobacterium tuberculosis* strains from the Beijing and Manila families account for the majority (~70%) of tuberculosis cases in Hawaii (http://health.hawaii.gov/tb/data-statistics/). There appears to be an ethnic association between the Beijing family and northern Asians and the Manila family and Filipinos. The Beijing family isolate (*M. tuberculosis* 96075) was collected in 1995 from a 44-year-old man living in Beijing, China (2). The isolate was sensitive to the first-line antibiotics isoniazid, rifampin, streptomycin, and ethambutol. Spoligotyping indicated that it was the Beijing type (shared type 1, octal code 000000000003771). The Manila family isolate (*M. tuberculosis* 96121) was collected in 1996 from a female patient living in Manila, Philippines (3). Spoligotyping indicated that it was the Manila type (shared type 19, octal code 677777477413771). Beijing family belongs to lineage 2, and the Manila family belongs to lineage 1 (J. T. Douglas, unpublished data). In addition, they are separated into modern and ancient lineages, respectively, and are members of the principal genetic group 1 of tuberculosis (4). These two complete genomes will serve as reference genomes for more detailed investigations of these important evolutionary lineages.

DNA was extracted with an overnight lysozyme proteinase K lysis procedure, a 24:1 chloroform-isoamyl alcohol separation, followed by ethanol precipitation. Primary genome sequencing was conducted using Roche 454 pyrosequencing at the Advanced Studies in Genomics, Proteomics, and Bioinformatics (ASGPB) Genomics Laboratory, University of Hawaii, Manoa. Hawaii. Genome finishing was done using hybrid assemblies of Roche 454 8-kb paired-end reads and Illumina GAIIx reads, and then it was closed using Sanger sequencing. Each genome was initially sequenced to ~25× coverage using Roche 454. The initial genome assembly for the 454 data was performed using Newbler 2.6 (Roche). The Illumina GAIIx reads (>100× coverage) were mapped to the scaffolds using the Burrows-Wheeler Aligner (BWA). Sequence gaps were filled through Sanger sequencing of the PCR products.

Genome annotation was done on the NCBI Prokaryotic Genome Annotation Pipeline (PGAP) version 2.0 (http://www.ncbi.nlm.nih.gov/genome/annotation_prok/). Annotations with Rapid Annotation using Subsystem Technology (RAST) version 4.0 (5) and Prokka (6) identified higher numbers of genes, but only the PGAP results are shown here. For Beijing 96075, PGAP predicted 3,981 protein-coding genes in the 4,379,376-bp genome. For Manila 96121, PGAP predicted 3,994 protein-coding genes in the 4,410,945-bp genome.

### Nucleotide sequence accession numbers.

These two whole genomes were deposited in GenBank under the accession numbers CP009426 (Beijing strain 96075) and CP009427 (Manila strain 96121).
